# Early Recognition and Management of Ocular Manifestations of Toxic Epidermal Necrolysis in a Pediatric Patient: A Case Report

**DOI:** 10.7759/cureus.70323

**Published:** 2024-09-27

**Authors:** Fatema K Abulfateh, Yazeed J AlHaqbani, Abdulrahman S Albuainain

**Affiliations:** 1 Ophthalmology, Bahrain Defence Force Hospital, Riffa, BHR

**Keywords:** amniotic membrane, drug-induced, mucocutaneous reaction, ocular manifestations, pediatric, prokera, stevens-johnson syndrome, toxic epidermal necrolysis

## Abstract

Toxic epidermal necrolysis (TEN) is a severe, often drug-induced mucocutaneous reaction characterized by widespread epidermal necrosis and detachment. Ocular involvement is common and can lead to significant morbidity if not addressed promptly. This case report presents a 10-year-old female with fever, rash, and severe ocular symptoms. Early use of Prokera, an amniotic membrane device, was instrumental in managing her ocular condition. After discharge and follow-up care, the patient showed marked improvement. This case highlights the importance of early recognition and intervention in ocular manifestations of TEN to prevent long-term complications.

## Introduction

Toxic epidermal necrolysis (TEN) and Stevens-Johnson syndrome (SJS) are severe, life-threatening mucocutaneous reactions characterized by extensive epidermal necrosis and detachment. Early diagnosis and treatment of these conditions are crucial, as they are often triggered by adverse drug reactions. Common culprits include antibiotics like penicillins, cephalosporins, sulfonamides, and fluoroquinolones; anticonvulsants such as phenytoin, lamotrigine, and phenobarbital; and nonsteroidal anti-inflammatory drugs (NSAIDs) like ibuprofen and naproxen. SJS is characterized by skin detachment involving less than 10% of the body surface area, while TEN involves more than 30% of the total body surface area [1-4].

Ocular involvement occurs in approximately 50% of TEN/SJS cases and can lead to complications ranging from mild conjunctivitis to severe vision loss due to corneal scarring and symblepharon formation. Common late complications include severe dry eyes and trichiasis [5-7]. Timely and aggressive management of ocular symptoms is essential to prevent long-term sequelae. Prokera, a therapeutic device made from cryopreserved amniotic membrane, has shown efficacy in promoting healing and reducing inflammation of the ocular surface in SJS/TEN patients [8,9]. This case report details the clinical course and management of TEN in a pediatric patient, emphasizing the significant role of Prokera in treating acute ocular manifestations.

## Case presentation

A 10-year-old girl with a history of heavy menstrual periods and mild eczema managed with tranexamic acid, dydrogesterone, and NSAIDs began experiencing a sore throat and cough four days prior to her ER visit. For her symptoms, she took ibuprofen, paracetamol, and antihistaminic cough syrup. The day before her ER visit, she developed a fever of 39 °C and sudden onset of extensive indurated erythema on her face and body, accompanied by erosion of the lips and inner buccal mucosa. This led her to visit two different health centers, where she received hydrocortisone and paracetamol without improvement. On the day of her ER presentation, she noticed swelling of her lips, difficulty swallowing, redness, discharge, and blurred vision in both eyes, and her fever worsened to 40 °C.

Upon admission, the patient was lethargic, with swollen eyelids, conjunctivitis with purulent discharge, and swollen lips with blisters. Erythema and erosions were present on her face and body, along with a few petechial spots on the abdomen and vasculitis-type lesions. The estimated body surface area involvement was around 25%, suggesting SJS/TEN overlap. Vital signs indicated septic shock: temperature 40 °C, heart rate 132 bpm, respiratory rate 42 breaths/min, blood pressure 91/53 mmHg, and oxygen saturation 88%, improving to 93% with supplemental oxygen. Initial management included intravenous fluids for dehydration, intravenous meropenem (800 mg every eight hours), intravenous methylprednisolone, and intravenous immunoglobulin (IVIG). The differential diagnoses considered included sepsis, anaphylaxis, and SJS/TEN overlap, with sepsis as the primary concern due to vital sign abnormalities. IVIG was administered early due to significant mucosal involvement, addressing the risk of SJS/TEN, while broad-spectrum antibiotics were initiated promptly. Cultures were pending to determine any underlying etiology.

Upon ophthalmology consultation, no ocular manifestations were noted except for swollen eyelids and conjunctivitis with purulent discharge. Prokera lenses were applied bilaterally before the development of any ocular surface manifestations as part of the management of the inflammatory response and to promote healing if ocular issues arose. The treatment regimen also included prednisolone acetate 1% eye drops every three hours, ofloxacin eye drops 0.3% four times a day, carboxymethylcellulose sodium 0.5% eye drops every 30 minutes, tobramycin 0.3%/dexamethasone 0.1% eye ointment applied four times a day over the eyelids and lashes, and eyelash hygiene every two hours using wet cotton tips for both eyes.

Over the course of her inpatient stay, the patient’s condition fluctuated, with episodes of desaturation, fever spikes, and hemodynamic instability. This necessitated intensive monitoring and support, including high-flow nasal cannula oxygen, central venous line placement, and adjustments in medication dosages based on her clinical status and laboratory results.

**Figure 1:**
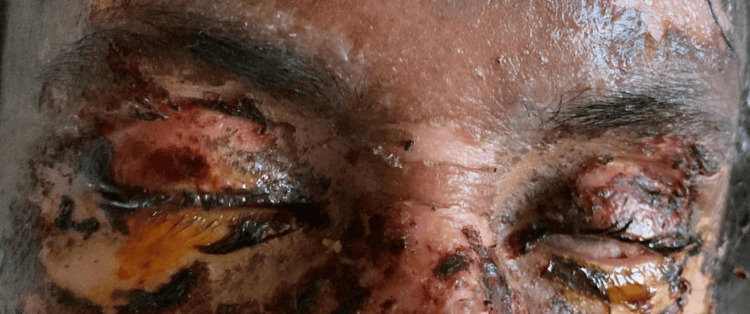
Eyelid skin sloughing, revealing a portion of the bulbar conjunctiva with congestion and an epithelial defect

During her hospital admission, the patient progressed from SJS/TEN overlap to TEN within the first week; the estimated surface area involvement increased to around 35%. Her laboratory results initially showed leukopenia with a count of 1,640 and neutrophil predominance (88.5%), along with elevated CRP of 88 mg/dl and erythrocyte sedimentation rate of 50 mm/hr. The patient exhibited sloughed eyelid skin and a bulbar conjunctival epithelial defect in both eyes (Figure 1). The frequency of prednisolone acetate 1% eye drops was increased to every hour, ofloxacin eye drops 0.3% were replaced with moxifloxacin 0.5% eye drops four times a day, and cold compresses were applied four times a day to both eyes.

On day 10 of admission, the patient developed a corneal epithelial defect (CED) measuring 4 × 4 mm in both eyes. Prokera was removed and replaced with a new one in each eye. Additionally, upper and lower fornices were swept with a cotton tip. Further medications were added to her previous daily treatment plan: tobramycin 0.3%/dexamethasone 0.1% eye ointment for both eyes and oral vitamin C at 1 gram once daily, as tolerated. Prednisolone acetate 1% eye drops were decreased to every three hours.

During daily reviews, lubricating eye drops were gradually reduced to every three hours, while polyacrylic acid 0.2% gel was added every four hours, resulting in a decrease in the CED size to 3 × 3 mm. By day 15, methotrexate 7.5 mg and folic acid 5 mg were added once weekly, and a complete blood count was requested to monitor for contraindications and side effects related to methotrexate.

On day 16, the CED showed improvement. The Prokera for the right eye was partially dissolved, while the left eye’s Prokera completely dissolved, leading to its removal. Prednisolone acetate 1% eye drops were decreased to every four hours for both eyes. The next day, the right eye’s Prokera was also removed, and the CED worsened in the right eye to 4 × 4 mm, while the left eye improved to 2 × 2 mm. The patient continued the same medication regimen.

By day 18, the CED in the right eye improved to 3 × 3 mm, while the left eye remained at 2 × 2 mm. Oral prednisolone was prescribed in tapering doses, and prednisolone acetate 1% eye drops were decreased to four times a day. The following day, the CED in the right eye measured 1 × 1 mm, and the left eye had healed completely. The patient was discharged from the hospital with follow-up care scheduled in three days. Instructions included regular complete blood counts and liver function tests to monitor for methotrexate toxicity, intraocular pressure (IOP) checks due to steroid use, and management of ocular surface disease with lubrication and anti-inflammatory medications.

During the first follow-up visit three days after discharge, the patient’s medications included prednisolone acetate 1% four times a day (QID), moxifloxacin 0.5% four times a day (QID), carboxymethylcellulose sodium 0.5% every four hours, polyacrylic acid 0.2% gel every four hours, methotrexate 7.5 mg weekly, and folic acid 5 mg weekly. Examination revealed no stain uptake on the conjunctiva and cornea in both eyes. The plan included monitoring CBCs, tapering oral prednisolone, tapering moxifloxacin twice a day (BID), maintaining prednisolone acetate 1% QID, and continuing eye hygiene.

In subsequent follow-ups, the patient’s condition was assessed regularly. Findings included fornices without symblepharon, confluent superficial punctate keratitis on the cornea, early lid keratinization, and conjunctival scarring. The treatment plan involved increasing methotrexate to 10 mg weekly, adding white petrolatum and mineral oil (Duratears) four times a day, increasing lubricant drops to every two hours, and applying bandage contact lenses (BCLs) in both eyes while continuing other medications.

As follow-ups continued, the patient's visual acuity was recorded at 6/18 in both eyes. Eyelid and conjunctival keratinization were noted, with BCLs in place. Elevated IOP (30 mmHg) was measured in both eyes. Prednisolone acetate (1%) was discontinued, and additional medications such as dorzolamide 2%, timolol 0.5% twice daily, and loteprednol etabonate 0.5% every six hours were initiated to manage IOP and inflammation, respectively.

Two days later, the patient developed bilateral eyelid thickening and a CED of 2 × 2 mm in the right eye, attributed to rubbing eyelashes. The BCL was replaced, and the rubbing eyelashes were removed. Cyclosporine 0.05% was added for its anti-inflammatory and lubrication properties.

Four days later, the right CED healed with subsequent scarring (2 × 4 mm), and eyelid margins remained thickened without entropion or ectropion. There were no rubbing lashes in both eyes, and a good red reflex was observed. Tobramycin 0.3%/dexamethasone 0.1% ointment was added around the eyes twice daily, and the methotrexate dose was increased to 12.5 mg.

On her next visit, the IOP measured 21 mmHg in the right eye and 22 mmHg in the left eye. The dorzolamide 2% and timolol 0.5% were changed to brimonidine 0.15% three times a day, and the tobramycin 0.3%/dexamethasone 0.1% ointment was replaced with fusidic acid viscous (Fucithalmic). The BCL was routinely exchanged between visits.

An oculoplastic consultant noted improved eyelid edema, but there were still rubbing eyelashes, which were removed. No superior or inferior fornix shortening, entropion, or ectropion was observed. Loteprednol etabonate 0.5% was tapered twice daily, and tacrolimus ointment 0.01% was introduced for application inside the eyes due to its immunomodulatory effects on severe mucocutaneous involvement.

At a follow-up 10 weeks later, the patient developed a corneal infiltrate in the right eye measuring <1 mm with stain uptake, which was treated with high-frequency moxifloxacin 0.5% with slow tapering, taking about two months to resolve. The IOP was recorded at 28 mmHg in the right eye and 27 mmHg in the left eye, requiring the resumption of dorzolamide 2% and timolol 0.5%.

**Figure 2:**
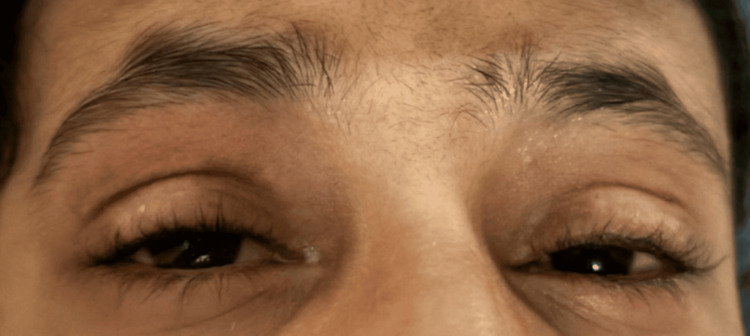
Significant improvement in ocular surface healing, with stabilization of conjunctival and corneal conditions The left and right eyelids appear normal, and the conjunctiva is quiet.

The final outcome demonstrated significant improvement in ocular surface healing and stabilization of conjunctival and corneal conditions (Figure 2). The patient’s long-term medication regimen included dorzolamide/timolol (Cosopt), moxifloxacin (Vigamox), lubrication, cyclosporine (Restasis), tacrolimus, methotrexate, folic acid, and fusidic acid ointment with hydrocortisone acetate. The patient was advised to avoid penicillins, cephalosporins, and NSAIDs due to the suspected drug-induced SJS. Ongoing follow-up visits were recommended for monitoring and prompt reporting to the ER if necessary.

## Discussion

Ocular involvement in TEN can lead to severe complications, including corneal ulceration, scarring, and blindness if not managed promptly and effectively [4,7]. Prokera, which consists of a cryopreserved amniotic membrane, has been shown to reduce inflammation, promote epithelial healing, and inhibit fibrosis [10]. As an FDA-approved therapeutic device, Prokera is utilized to treat various ocular surface diseases, including the acute phase of TEN/SJS, due to its natural anti-inflammatory, anti-scarring, and anti-angiogenic properties [11].

Topical corticosteroids and antibiotics are commonly employed to reduce inflammation and prevent secondary infections, respectively. While effective in controlling acute inflammation, these treatments do not address the underlying tissue damage and are insufficient in preventing scarring and chronic ocular surface issues [12]. Amniotic membrane transplantation (AMT), including Prokera, has demonstrated superior outcomes in managing acute ocular manifestations of TEN/SJS. The properties of Prokera lead to rapid symptom relief, promotion of epithelial healing, and a reduction in the risk of long-term complications such as corneal scarring and symblepharon formation [12,13].

Multiple studies have shown that patients treated with Prokera during the acute phase of TEN experienced faster resolution of ocular surface inflammation, resulting in improved outcomes, reduced pain, accelerated healing of epithelial defects, lower rates of long-term ocular surface complications, and fewer chronic complications compared to those receiving conventional treatment alone [14-17]. Gregory emphasized that early intervention with AMT significantly decreased the incidence of severe visual impairment and chronic ocular surface disease in TEN patients [12].

The amniotic membrane in Prokera acts as a biological bandage, providing a protective environment for the ocular surface. It contains essential growth factors and cytokines that promote tissue regeneration, reduce inflammation, and prevent fibrosis, which is critical in managing both the acute and chronic phases of TEN [18]. Tseng et al. and Meller et al. demonstrated that the amniotic membrane can significantly improve ocular outcomes in TEN/SJS patients by promoting epithelialization and reducing fibrosis [19,20].

The comprehensive management approach for this patient included systemic corticosteroids, IVIG, immunomodulator therapy (methotrexate, topical tacrolimus, and cyclosporin), antibiotics, and supportive care. Systemic corticosteroids and IVIG are commonly utilized to manage severe TEN/SJS by modulating the immune response and reducing inflammation [3]. The combination of these treatments, alongside the application of Prokera, significantly contributed to the patient’s recovery.

The patient’s clinical course was marked by fluctuating symptoms, necessitating intensive monitoring and support. Close monitoring and timely treatment adjustments were critical for managing complications and ensuring optimal outcomes. This underscores the importance of a multidisciplinary approach and frequent reassessment in managing severe TEN/SJS cases [21,22]. Gueudry et al. (2009) emphasized the necessity of early intervention and close monitoring to prevent long-term ocular complications [5].

Managing TEN in pediatric patients presents unique challenges, such as dosing adjustments and the impact on growth and development. A multidisciplinary approach involving dermatology, ophthalmology, and critical care is essential to address the diverse complications [23]. Follow-up care in dermatology and ophthalmology is crucial for monitoring potential long-term complications and managing residual symptoms. Regular follow-ups ensure early detection and treatment of sequelae, thereby improving long-term outcomes [21-23].

The outcomes of this case align with existing literature on the use of Prokera in pediatric TEN patients. Studies have reported significant improvements in ocular surface healing and reductions in complications associated with AMT [24]. Prokera should be considered for use in SJS and TEN within the first week after the onset of ocular symptoms. Early intervention is vital to reduce inflammation, promote epithelial healing, and prevent scarring or other long-term complications [25]. This case contributes to the growing body of evidence supporting the efficacy of Prokera in managing TEN-related ocular manifestations.

Biologics, particularly etanercept, have shown promise in treating SJS/TEN, demonstrating lower mortality, fewer complications, and shorter hospitalization times compared to other treatments. Although not statistically significant, etanercept may offer a survival benefit. Further studies are needed to confirm its efficacy and safety [26].

Further research is necessary to validate the efficacy of Prokera in pediatric TEN patients through larger studies. Developing standardized treatment protocols for managing ocular manifestations of TEN/SJS could guide clinicians and improve patient outcomes. Continued exploration of novel therapeutic approaches and their integration into clinical practice is essential.

## Conclusions

This case underscores the critical importance of early intervention with Prokera in managing ocular manifestations of TEN in pediatric patients. The application of Prokera, in conjunction with systemic and supportive treatments, was vital in preventing long-term ocular complications. Ongoing research and heightened clinical awareness are essential to enhance outcomes and refine care strategies for pediatric TEN with ocular involvement.
